# Increased Plasma Dipeptidyl Peptidase-4 Activities in Patients with Coronary Artery Disease

**DOI:** 10.1371/journal.pone.0163027

**Published:** 2016-09-21

**Authors:** Guang Yang, Yuzi Li, Lan Cui, Haiying Jiang, Xiang Li, Chunzi Jin, Dehao Jin, Guangxian Zhao, Jiyong Jin, Rui Sun, Limei Piao, Wenhu Xu, Chenghu Fang, Yanna Lei, Kuichang Yuan, Chunhua Xuan, Dazi Ding, Xianwu Cheng

**Affiliations:** 1 Department of Cardiology, Yanbian University Hospital, Yanji, Jilin P.R., China; 2 Department of Physiology, Yanbian University Medical College, Yanji, China; 3 Department of Central Laboratory, Yanbian University Hospital, Yanji, China; 4 Department of Angiography Center, Yanbian University Hospital, Yanji, China; 5 Department of Internal Medicine, Kyung Hee University, Seoul, South Korea; Heart Research Institute, AUSTRALIA

## Abstract

Dipeptidyl peptidase-4 (DPP4) is one of the most potent mammalian serine proteases participated in the pathogenesis of subclinical atherosclerosis. Here we investigated whether the plasma soluble form of DPP4 is associated with the prevalence of coronary artery disease (CAD) with and without diabetes mellitus (DM). A cross-sectional study was conducted of 496 aged 26–81 years with (n = 362) and without (n = 134) CAD. Plasma DPP4 activity, high sensitive C-reactive protein (hs-CRP), low-density lipoprotein cholesterol (LDL-C), and high-density lipoprotein levels were measured. The coronary atherosclerotic plaques were evaluated by coronary angiography. The CAD patients with (n = 84) and without (n = 278) DM had significantly higher DPP4 levels (11.8 ± 3.1 vs. 6.9 ± 3.5 ng/mL, *P*<0.01) than the nonCAD subjects. The acute coronary syndrome patients (n = 299) had elevated DPP4 levels than those with stable angina patients (n = 83). CAD patients even without DM had increased plasma DPP4 activities as compared with nonCAD subjects (10.9 ± 4.9 vs. 6.4 ± 3.1, ng/L, P< 0.01). A linear regression analysis revealed that overall, the DPP4 levels were positively associated with LCL-C and hs-CRP levels as well as syntax scores. A multiple logistic regression analysis demonstrated that plasma DPP4 activity was independent predictor of CAD (odds ratio, 1.56; 95% CI, 1.19–1.73; *P*<0.01). Our study shows that increased DPP4 activity levels are associated with the presence of CAD and that the plasma DPP4 level serves as a novel biomarker for CAD even without DM.

## Introduction

In the initial years after its discovery, dipeptidyl peptidase-4 (DPP4) (also known as T-cell activation antigen CD26 or adenosine deaminase complexing protein 2 [ADCP2]) was considered to be a serine exopeptidase belonging to the S9B protein family and to function to remove X-proline dipeptides from the N-terminus of polypeptides (e.g., neuropeptides, neurohormones, and inflammatory chemokines) in the extracellular space [1]. Over the last five years, emerging data revealed unexpected roles for DPP family members in intracellular signaling, lipid metabolism, oxidative stress production, immune activation, insulin resistance, and inflammation [2–7]. These activities confer a broad range of molecular functions on the DPP family, with clinical implications for a potential pathological role in inflammatory and metabolic diseases [8–11].

Among the eight DPPs known family members, DPP4, which is one of the most potent serine peptidases, was reported to show widespread expression in mammalian tissues, including small intestine, kidney, liver, adipose, and heart tissues [12]. DPP4 presents in the circulatory system and body fluids as a soluble form, in which the extracellular domain of the molecule is thought to be produced by proteolytic cleavage from the cell surface [13]. The results of studies using colorimetric enzyme immunohistochemistry revealed that DPP4 protease activity is localized in the capillary endothelia of rats and human heart tissues [10,13,14]. DPP4 activity has obtained great interest as a therapeutic target, and DPP4 inhibitors that elongate the insulinotropic effect of glucagon-like peptide-1 (GLP-1) are now widely used as antidiabetic drugs [3]. Accumulating evidence shows that the beneficial actions of DPP4 inhibition (i.e., antiapoptosis, antioxidative stress, and anti-inflammation) are most likely partially responsible for the multiple pleotropic effects targeting the metabolic and atherosclerosis-based cardiovascular disorders addressed by this class of agents [2–5,8,15–17]. Experimental and clinical studies revealed that inflammatory states including diabetes mellitus (DM), obesity, and atherosclerosis show increased plasma DPP4 levels [10,18,19]. In addition, in cancer biology research, circulating DPP4 activity was demonstrated to be a therapeutic target and biomarker for several cancers [20,21]. These findings led us to speculate that increased plasma DPP4 activity may be associated with atherosclerosis-related vascular disease. However, until now it is unclear regarding the association between circulating DPP4 activity and coronary artery disease (CAD) in individuals with or without DM.

Consequently, in this study, we examined whether plasma DPP4 activity levels are associated with the presence of CAD even in patients without DM. Our results demonstrate that high DPP4 activity levels are independently associated with the prevalence of CAD even after adjustment for the known CAD risk factors.

## Materials and Methods

### Study population and definition

This study protocol was approved by the Ethics Committee of Yanbian University Hospital, and written informed consent was obtained from all patients. We screened a total of 362 consecutive patients referred for percutaneous coronary intervention (PCI) with drug-eluting stent implantation from May 2014 to December 2015 at Yanbian University Hospital. The CAD patients were sub-grouped into the acute coronary syndrome (including the acute myocardial infarction [AMI] and the unstable angina pectoris [UAP]; n = 299) and the stable angina pectoris (SAP; n = 63) groups based on their symptoms and clinical examination marks [22,23]. According to the presence/absence of DM, we also divided the CAD patients into CAD/DM(+) and CAD/DM(-) groups. The 134 age-matched control subjects were also divided into nonCAD/DM(+) and nonCAD/DM(-). A total of 134 individuals who no significant luminal narrowing of the coronary vessels by a coronary angiography (CAG), no evidence of coronary artery problem (confirmed as no myocardial infarction [MI] by history, no typical symptom on exertion, electrocardiogram, and negative exercise test) were considered the nonCAD subjects. Patients were excluded if they had prior evidence of congenital heart disease, primary valvular disease, cardiomyopathy, secondary cardiac muscle disease caused by any known systemic condition, or end-stage kidney disease. The patients with DM who were taking a DPP4 inhibitor were also excluded.

SAP was defined if patients had an invariable exertional chest pain for over the 3 months prior to the patient’s admission to the hospitalization (‘invariable’ indicating the same grade of excitation provocation and exertion and the same quality, quality, and 3- to 5-min continuance, lighted by nitroglycerin rest [24]. We diagnosed UAP by typical symptom at rest in the 24 hr before the patient was admitted to the hospitalization, T-wave inversion on electrocardiogram and/ro depressed ST ≥0.1 mV but a normal creatine kinase-MB level. AMI was defined if patients had a history of prolonged typical symptom, an ECG indicative of new ischemia (new left bundle branch block or new ST-T change) and a elevated biomarker (at least one positive biomarker: troponin T or creatine kinase-MB) [25].

In the study, dyslipidemia was defined as total cholesterol ≥ 220 mg/dL or lowdensity lipoprotein cholesterol ≥140 mg/dL or previously known dyslipidemia. DM was defined if patients had a history of any anti-diabetic drug, previously known DM, a fasting plasma glucose concentration >126 mg/dL, HbA1c levels ≥ 6.5% or/and a random plasma glucose concentration of >200 mg/ dL [26]. Smoking status was confirmed as positive if patients were had stopped smoking within 6months before checking coronary or smoking currently. Hypertension was diagnosed as diastolic blood pressure ≥ 90 mmHg and/or systolic blood pressure ≥ 140 mmHg, or previously known, or medication-dependent [27].

A standard questionnaire was administered by two observers, recording demographic characteristics including age, gender, smoking history, medication history, and clinical history (hypertension, diabetes, previous MI, previous PCI, previous bypass surgery, and previous cerebrovascular disease). The measurement of body weight index (BMI) and blood pressure were as described [23]. Venous blood samples were obtained prior to PCI and stored at –80°C.

### Plasma DPP4 activity analysis

We measured each subject’s DPP4 activity by using the DPP4 Glo Protease Assay (Promega, Madison, WI) with an aminoluciferin substrate. The luminogenic substrate contains the Gly-Pro sequence recognized by DPP4. Following DPP4 cleavage, the substrate for luciferase (aminoluciferin) is released, resulting in the luciferase reaction and the production of light. For the blood DPP4 activity assays, the plasma was isolated using VENOJectII vacuum blood collection tubes containing anticoagulants without a serine protease inhibitor (Terumo, Tokyo), and the plasma was then diluted in 0.1 mM Tris-HCl buffer (pH 8.0) by 30-fold. Equal amounts of diluted plasma (25 μl) were subjected to a DPP4 Glo assay (Promega) in the presence or absence of the DPP4 inhibitor anagliptin (20 μmol/L). Human recombinant DPP4 (Sigma-Aldrich) was used to drive a standard curve. The luminescence intensity was calculated using a luminometer, and we use the anagliptin-sensitive value in relative light units (RLUs) per μl of plasma to represent the DPP4 activity (ng/L). The inter-assay coefficient of variation was 4.7 and the intra-assay coefficient of variation was 7.0%.

### Quantitative coronary angiogram (QCA)

The quantitative coronary angiogram (QCA) was evaluated from angiography exhibiting the maximal degree of stenosis. We did the QCA analysis using a contour detection minimum cost algorithm (DSA Artis Zee Biplane; Siemens, Erlangen, Germany). Patients with CAD had severe stenosis defined as the presence of ≥50% diameter stenosis of at least one major artery. The reference segment diameter was obtained the averages from 5-mm long angiographically normal segments proximal to the lesion; if a normal proximal segment could not be identified, a distal angiographically normal segment was analyzed as described [28].

### Laboratory examination

Each subject’s plasma levels of hemoglobin A1c (HbA1c), high sensitive C-reactive protein (hs-CRP), low-density lipoprotein cholesterol (LDL-C), creatinine, and high-density lipoprotein cholesterol (HDL-C) were examined at the clinical laboratory of Yanbian University Hospital (Clinical Laboratory, Yanji, China) [29].

### Statistical analysis

All of the statistical analyses were performed using SPSS 16.0 software (SPSS, Chicago, IL). Normally distributed data are expressed as the mean ± standard deviation (SD), whereas variables with a skewed distribution were log transformed to approximate normality before analysis. Categorical variables are expressed as the frequency or percentage. The chi-square test was used to compare the categorical variables among the study groups. We used a one-way ANOVA (for the comparisons of continuous parameters among three or more groups), followed by a Tukey post hoc test to analyze significant differences or Student’s *t-*test (for the comparisons of continuous parameters between two groups). Associations between continuous variables were tested by partial correlation analyses. The elements that were linked at the *P*<0.05 level were isolated by a univariate analysis as independent suitable candidates for a multiple regression analysis, which was applied to study the independent contributing factors to CAD. We used a linear regression analysis to calculate the correlation coefficients. In all statistical analyses, two-sided *P-*values <0.05 were considered significant.

## Results

### Patients’ laboratory and clinical characteristics

The baseline characteristics of the CAD subjects (n = 362) and nonCAD subjects (n = 134) were presented in Table 1. There was a significant difference in the gender distribution of two experimental groups, but no significant between-group difference in age or BMI.

**Table 1:** Baseline characteristics.

	CAD (n = 362)	nonCAD (n = 134)	*P* value
Age, yrs	61.7 ± 10.1	54.9 ± 11.0	0.18
Female, %	33.0	54.0	< 0.01
BMI, kg/m2	25.4 ± 13.8	25.1 ± 3.7	0.55
Clinical histories			
Hypertension, %	63.3	47.8	< 0.01
Diabetes mellitus, %	23.2	16.4	0.08
Current smokers, %	51.0	33.0	< 0.01
Previous MI, %	13.0	0.0	< 0.01
Previous PCI, %	13.0	0.0	< 0.01
Previous bypass surgery, %	1.0	0.0	0.08
Previous cerebrovascular disease, %	13.0	0.0	< 0.01
Blood Examination			
LDL-C, mg/dl	102.3 ± 31.2	93.8 ± 28.9	0.31
HDL-C, mg/dl	43.8 ± 19.5	47.4 ± 28.5	0.18
Hemoglobin A1c, %	6.0 ± 1.5	5.5 ± 1.1	0.01
Creatinine, mg/dl	1.0 ± 0.3	0.9 ± 0.1	0.61
hs-CRP, mg/dl	15.2 ± 28.9	3.3 ± 9.0	0.04
DPP4, ng/L	11.8 ± 3.1	6.9 ± 3.5	0.008
Medications			
ARBs or ACEIs, %	39.0	23.0	< 0.01
CCBs, %	23.7	17.2	0.001
β-blockers, %	64.8	35.3	0.96
Anti-lipids, %	98.3	87.3	< 0.01
Aspirin, %	98.9	98.5	0.53
Insulin, %	10.3	6.0	< 0.01

Values are expressed as mean ± SD or number (%). BMI, body mass index; MI, myocardial infarction; PCI, percuteneous coronary intervention; LDL-C, low density lipoprotein cholesterol; HDL-C, high density lipoprotein cholesterol; hs-CRP, high-sensitive c-reactive protein; DPP4, dipeptidyl peptidase-4; ACEI, angiotensin converting enzyme inhibitor; ARBs = Angiotensin II receptor blockers; CCBs, calcium channel blockers.

The CAD patients showed a significantly higher prevalence of hypertension; these patients also showed a significantly higher numbers of current smokers and to have had cerebrovascular disease or a previous MI, or to have undergone PCI (*P*>0.05 for comparison). The frequencies of the CAD patients under intervention with anti-diabetic (insulin), anti-platelet, anti-lipid, and antihypertensive drugs were all higher than those of the nonCAD subjects.

Based on their symptoms and clinical examination results, the CAD patients were sub-grouped into UAP+AMI and SAP groups. The laboratory and clinical characteristics of the UAP+AMI and SAP groups are presented in Table 2. There were no significant differences in age or BMI between the SAP and UAP+AMI groups (*P*>0.05 for each comparison). With the exception of the prevalence of DM and previous cerebrovascular disease, there was no significant difference in the medication use and clinical histories between both experimental sub-groups (*P*>0.05 for each comparison).

**Table 2:** Demographic and Clinical Variables of SAP and UPA+AMI.

	SAP (n = 63)	UAP+AMI (n = 299)	*P* value
Age, yrs	60.4 ± 8.9	62.0 ± 10.4	0.27
Female, %	44.0	30.0	0.04
BMI, kg/m2	24.4± 2.9	25.6 ± 15.1	0.51
Clinical histories			
Hypertension, %	65.1	62.9	0.74
Diabetes mellitus, %	12.7	25.4	0.01
Current smokers, %	48.0	51.0	0.61
Previous myocardial infarction, %	13.0	13.0	1.00
Previous PCI, %	18.0	13.0	0.35
Previous bypass surgery, %	0.0	1.0	0.52
Previous cerebrovascular disease, %	7.0	14.0	0.04
Blood Examination			
LDL-C, mg/dl	95.5 ± 29.4	104.0 ± 31.4	0.04
HDL-C, mg/dl	45.0± 21.4	43.7 ± 19.0	0.64
Hemoglobin A1c, %	5.6 ± 1.0	6.1 ± 1.5	0.01
Creatinine, mg/dl	73.7 ± 23.4	82.1 ± 44.5	0.15
hs-CRP, mg/dl	3.0 ± 7.2	17.8 ± 31.0	< 0.01
DPP4, ng/L	9.4 ± 3.8	13.1 ± 4.2	< 0.01
Medications			
ARBs or ACEIs, %	37.0	39.0	0.70
CCBs, %	20.0	25.0	0.41
β-blockers, %	54.0	67.0	0.07
Anti-lipids, %	97.0	99.0	0.43
Aspirin, %	100	99.0	0.36
Insulin, %	7.0	11.0	0.24
Target lesion location			
Left main artery	1	12	0.35
Left anterior descending artery	45	257	0.02
Left circumflex artery	8	187	< 0.01
Right coronary artery	17	192	< 0.01
QCA of target lesions			
Reference vessel diameter, mm	3.4 ± 2.4	2.7 ± 0.8	0.42
Diameter stenosis, %	74.3 ± 7.1	87.3 ± 9.2	0.04
Lesion length, mm	15.0 ± 4.4	19.1 ± 4.0	< 0.01
Stent types			
Firebird	40.9	59.1	0.16
Lepu	40.4	40.9	0.68
Syntax score	11.9 ± 6.3	17.8 ± 7.9	< 0.01

QCA, quantitative coronary angiography; other abbreviations are as Table 1. Values are expressed as mean ± SD or number (%).

### Atherosclerotic lesion characteristics

As presented in Table 2, although there was no significant difference in the target lesion locations of the left main artery (*P*>0.05 for each analysis), the UPA+AMI group had a higher ratio of target lesion location in the left anterior descending artery, left circumflex artery, and right coronary artery compared to the SAP patients (*P*<0.05 for all comparisons). In the QCA analysis of target lesions, with the exception of reference vessel diameter (*P*>0.05 for each analysis), the patients with UAP or AMI had significantly greater diameter stenosis (87.3 ± 9.2 vs. 74.3 ± 7.1 mm, *P* = 0.04) and lesion length (19.1 ± 4.0 vs. 15.0 ± 4.4 mm, *P*<0.01) as well as Syntax score (17.8 ± 7.9 vs. 11.9 ± 6.3, *P*<0.01) compared to the patients with SAP (Table 2).

### Circulating Biomarkers

The CAD group had significantly increased plasma DPP4 levels (11.8 ± 3.1 vs. 6.9 ± 3.5 ng/L, *P*<0.01) compared to the nonCAD group (Table 1). The levels of hs-CRP (15.2 ± 28.9 vs. 3.3 ± 9.0 ng/mL, *P* = 0.04) and hemoglobin A1c (6.0 ± 1.5 vs. 5.5 ± 1.1%, *P*<0.01) were significantly higher in the CAD patients compared to the nonCAD subjects (*P*<0.01 for each comparison), but there was no significant difference in the levels of creatinine (1.0 ± 0.3 vs. 0.9 ± 0.1 mg/dl), LDL-C (102.3 ± 31.2 vs. 93.8 ± 28.9 mg/dl), or HDL-C (43.8 ± 19.5 vs. 47.4 ± 28.5 mg/dl) between the CAD and nonCAD groups (*P*>0.05 for each parameter analysis).

In the sub-analysis, the UAP or AMI patients had significantly increased levels of circulating DPP4 (13.1 ± 4.2 vs. 9.4 ± 3.8 ng/L, *P*<0.01). The UAP+AMI group patients had also significantly increased levels of plasma LDL-C (104.0 ± 31.4 vs. 95.5 ± 29.4 mg/dl, *P* = 0.04) and hs-CRP (17.8 ± 31.0 vs. 3.0 ± 7.2 ng/mL, *P*<0.01) and hemoglobin A1c (6.1 ± 1.5 vs. 5.6 ± 1.0%, *P* = 0.01) compared to the patients with SAP, whereas there was no significant difference in the HDL-C or creatinine levels between two experimental groups (*P*>0.05 for all comparisons).

### Impacts of diabetes and CAD on plasma DPP4 activities

DPP4 inhibitors are the most widely used incretin-based therapy for the management of type 2 DM globally [3]. A few recent clinical studies have demonstrated increased blood DPP4 activity in subclinical atherosclerosis (diabetes and obesity) [19]. We therefore sought to examine the impact of DM on blood DPP4 activity levels in patients with CAD in the present study. Our findings indicated that DM significantly increased plasma DPP4 activity in the nonCAD control subjects (8.0 ± 2.9 vs. 6.4 ± 3.1 ng/L, *P*<0.01), whereas it tended to increase the blood DPP4 activity levels in the CAD patients (12.2 ± 4.4 vs. 10.9 ± 4.9 ng/L, *P*>0.05) (Tables 3 and 4). With the exception of the levels of hemoglobin A1c and insulin treatment or/and BMI and hs-CRP, there were no significant differences in other laboratory and clinical parameters in the comparisons of the two experimental groups. Interestingly, we observed that CAD patients even without DM had increased plasma DPP4 activities as compared with nonCAD subjects (10.9 ± 4.9 vs. 6.4 ± 3.1, ng/L, P< 0.01), suggesting that CAD pathogenesis may also result in increase in plasma DPP4 activity.

**Table 3:** Demographic and Clinical Variables of nonCAD/DM(+) and nonCAD/DM(-).

	nonCAD/DM(+) (n = 22)	nonCAD/DM(-) (n = 112)	*P* value
Age, yrs	54.2 ± 9.0	55.0 ± 11.4	0. 75
Female, %	59.1	53.57	0.64
BMI, kg/m2	26.5 ± 3.3	24.8 ± 3.7	0.05
Clinical histories			
Hypertension, %	63.6	44.6	0.11
Diabetes mellitus, %	100	0	
Current smokers, %	20.0	35.14	0.15
Previous myocardial infarction, %	0.0	0.0	
Previous PCI, %	4.6	0.0	0.33
Previous bypass surgery, %	0	0	
Previous cerebrovascular disease, %	9.1	7.1	0.75
Blood Examination			
LDL, mg/dl	94.2± 24.4	93.7 ± 29.8	0.94
HDL, mg/dl	55.0 ± 50.7	46.0 ± 22.2	0.45
Hemoglobin A1c, %	6.9 ± 1.7	5.3 ± 0.7	< 0.01
Creatinine, mmol/L	64.0 ± 23.8	69.1 ± 20.0	0.29
hs-CRP, mg/dl	7.7 ± 18.1	2.4 ± 5.5	0.02
DPP4, ng/L	8.0 ± 2.9	6.4 ± 3.1	< 0.01
Medications			
ARBs or ACEIs, %	27.3	22.3	0.62
CCBs, %	13.6	17.9	0.63
β-blockers, %	50.0	32.4	0.12
Anti-lipids, %	90.9	86.6	0.58
Aspirin, %	100	98.2	0.53
Insulin, %	31.8	0.0	< 0.01

Abbreviations are as Table 1. Values are expressed as mean ± SD or number (%).

**Table 4:** Demographic and Clinical Variables of CAD/DM(+) and CAD/DM(-).

	CAD/DM(+) (n = 84)	CAD/DM(-) (n = 278)	*P* value
Age, yrs	61.5 ± 9.7	61.82 ± 10.3	0. 779
Female, %	44.1	29.5	0.019
BMI, kg/m2	25.8 ± 3.0	24.3± 3.6	< 0.01
Clinical histories			
Hypertension, %	73.8	60.1	0.02
Diabetes mellitus, %	100.0	0.0	
Current smokers, %	42.9	52.9	0.10
Previous myocardial infarction, %	13.4	13	0.92
Previous PCI, %	8.5	15.1	0.08
Previous bypass surgery, %	0.0	0.72	0.44
Previous cerebrovascular disease, %	16.1	12.3	0.39
Blood Examination			
LDL, mg/dl	105.3± 32.6	101.7 ± 29.8	0.37
HDL, mg/dl	42.4 ± 17.4	44.3± 19.9	0.45
Hemoglobin A1c, %	7.4 ± 1.7	5.6 ± 1.0	< 0.01
Creatinine, mmol/L	0.8 ± 0.4	0.7 ± 0.2	0.98
hs-CRP, mg/dl	12.0 ± 20.3	16.2 ± 31.0	0.16
DPP4, ng/L	12.2 ± 4.4	10.9 ± 4.9	0.06
Medications			
ARBs or ACEIs, %	40.5	38.9	0.79
CCBs, %	29.6	22.0	0.18
β-blockers, %	70.7	63.0	0.19
Anti-lipids, %	100	97.8	0.31
Aspirin, %	100	98.5	0.54
Insulin, %	43.9	0.0	

Abbreviations are as Table 1. Values are expressed as mean ± SD or number (%).

### Association between DPP4 activity and CAD patients with and without DM

We observed the DPP4 activities positively correlated with the levels of both LDL-C (*r* = 0.23, *P*<0.01) and hs-CRP (*r* = 0.41, *P*<0.01), whereas they were not correlated with the HDL-C levels (*r* = 0.05, *P*>0.05). The DPP4 activities were also positively correlated with the stenosis (*r* = 0.24, *P*<0.05) and lesion length (*r* = 0.19, *P*<0.05) shown by the CAG in patients with CAD.

As shown in Table 5, the logistic regression analysis revealed that CAD significantly associated with age, gender, hs-CRP, hypertension, LDL-C, and DPP4. The data from the multiple logistic regression analysis with gender and age as well as hs-CRP, LDL-C, hypertension, LDL-C, and DPP4 demonstrated that age (odds ratio [OR] 1.08; 95% confidence interval [CI] 1.05–1.11; *P*<0.01), gender (OR 0.29; 95% CI 0.17–0.49; *P*<0.01), hypertension (OR 3.65; 95% CI 1.98–5.78; *P*<0.01), and the levels LDL-C (OR 1.01; 95% CI 1.00 to 1.02; *P* = 0.02), hs-CRP (OR 1.06; 95% CI 1.02–1.11; *P*<0.01), and DPP4 (OR 1.56; 95% CI 1.19–1.73; *P*<0.01) were significantly correlated with CAD (Table 5).

**Table 5:** Independent predictors of CAD According to Multivariable Logistic Regression Analysis.

	Single			Multiple		
	Odds Ratio Estimate	95% CI	*P* value	Odds Ratio Estimate	95% CI	*P* value
Age (year)	1.06	1.04–1.085	< 0.01	1.08	1.05–1.11	< 0.01
Gender	0.41	0.27–0.613	< 0.01	0.29	0.17–0.49	< 0.01
BMI (kg/m^2^)	0.78	0.98–1.022	0.82			
Diabetes mellitus, %	1.54	0.92–2.583	0.053			
Hypertension, %	1.88	1.26–2.811	0.002	3.65	1.98–5.78	< 0.01
LDL cholesterol (mg/dl)	1.01	1.00–1.017	0.007	1.01	1.00–1.02	0.02
HDL cholesterol (mg/dl)	0.99	0.99–1.00	0.14			
hs-CRP, mg/dl	1.06	1.03–1.09	< 0.01	1.06	1.02–1.11	< 0.01
DPP4, ng/L	1.43	1.35–1.87	< 0.01	1.56	1.19–1.73	0.004

Multiple regression model includes all variables at baseline with P < 0.05 by univariable analysis. Abbreviations are as in Table 1. CI, confidence interval.

## Discussion

Previous experimental reports showing that DPP4 inhibition by genetic or pharmacological intervention alters vascular wall remodeling and atherosclerosis in mice [2,5,16,30] led us to hypothesize that serine protease DPP4 plays an important role in the initiation and progression of atherosclerosis. Limited information about is available regarding DPP4’s functions in humans, with the exception that plasma DPP4 activity has been shown to be increased in inflammation-related metabolic disorders (including obesity and diabetes) and carotid arterial atherosclerosis onset [18,19]. The results of the present study provide additional evidence to support the possible participation of DPP4 in atherosclerosis-based CAD with and without DM.

### DPP4 and inflammation/atherogenic lesion characterization

Accumulating evidence suggest that among the members if the DPP family, DPP4 is one of the important and abundant serine proteolytic enzyme synthesized by the blood cells and cardiovascular tissues, and that it is relevant to inflammation-associated metabolic disorders and their implications [3,8]. However, no previous study has examined the blood DPP4 concentrations in patients with or without CAD, to our knowledge. Our findings demonstrated the CAD patients had increased plasma DPP4 levels compared to the nonCAD subjects. The multivariable logistic regression analysis revealed that circulating DPP4 was independently associated with CAD. Because human metabolic states resulted in increased levels of DPP4 activity in the blood [18,19], we propose that elevated plasma sCD26 can use as a novel biomarker for CAD. Circulating DPP4 activity was recently targeted to treat patients with metastatic prostate cancer [21]. Pharmacological inhibition also mitigated injury-related neointimal formation and high fat diet-induced atherosclerosis in several animal models [5,16,30]. DPP4 is broadly distributed in mammalian tissues (i.e., small intestine, liver, adipose, kidney, heart tissues) [12]. A recent review noted that its multiple activities confer a broad range of functions of DPP4, with implications for potential pathophysiologic roles in metabolic and inflammatory disorders [3]. Thus, the inhibition of plasma DPP4 activity by DPP4 inhibitors could represent a common mechanism underlying their pleotrophic effects on inflammatory atherosclerosis-based cardiovascular disease.

CRP has been shown to be one of the acute-phase reactants underlying systematic inflammation, and that CRP exists predictive value for cardiovascular disorder or risk factors in healthy subjects [31,32]. Blood CRP can also be used to distinguish between unstable and unstable and stable coronary problem; e.g., patients with ACS had higher CRP levels compared to patients with SAP [31,33], and CAD patients had higher hs-CRP levels compared to those of nonCAD control subjects [33]. The proinflammatory effects of DPP4 have been partially addressed by clinical and experimental studies [34,35]. The positive correlation that we observed between DPP4 and hs-CRP supports our hypothesis that elevated levels of plasma DPP4 levels are associated with local inflammation within the arteries of patients with CAD. In the analysis of the subgroups of patients with CAD, we observed increased levels of DPP4 activity and hs-CRP in the UAP or AMI patients compared to patients with SAP. The analysis of the QCA of target lesions revealed that the UAP or AMI patients had higher values of diameter stenosis and lesion length as well as Syntax scores compared to the SAP group. In addition, the linear regression analysis revealed that in CAD patients, the DPP4 levels were also positively correlated with the stenosis and lesion length analyzed by the CAG. Collectively, these findings indicate that increased blood DPP4 levels provide important information on angiographic severity, the extent of inflammation and coronary artery disease.

### The impact of DM on plasma DPP4 levels in the CAD and non-CAD groups

A pair of recent clinical studies have described the clinical implications of elevated plasma DPP4 activity in conditions such as cerebral ischemia and osteoporosis [36,37]. Over the last five years, DPP4 inhibitors as a new class of anti-DM drugs have been widely used to manage patients with type 2 DM [2]. Consistent with a report that patients with inflammatory metabolic states such as diabetes had increased levels of blood DPP4 activity [3], we observed that the levels of plasma DPP4 were higher in the nonCAD/DM(+) subjects than in that of the control subjects, suggesting a potential role of DPP4 in the pathogenesis of metabolic disease and the management of this disease. It should be noted that our CAD patients with DM had a tendency to have increased DPP4 levels compared to the CAD-alone patients. In contrast to the nonCAD/DM(+) group patients, DM had no effect on the plasma hs-CRP levels in the patients with CAD. The lack of a significant effect might be in due part to the absence of a difference in the same extent of the inflammation between the CAD/DM(+) and CAD/DM(-) groups. Further investigations are needed to resolve this issue.

### DPP4 and lipid metabolism

We observed a positive correlation between the plasma DPP4 and LDL-C levels. LDL-C is recognized an critical factor in atherogenesis [38]. Lipoprotein uptake and metabolism by mainly macrophages in atherosclerotic plaque are critical pathologic steps in atherogenesis [39,40]. The relationship between hyperlipidemia and DPP4 activity has been studied extensively. Genetic or pharmacological intervention targeted toward DPP4 activity improved plasma dyslipidemia in mice [41,42]. DPP4 inhibition reduced postprandial lipemia as evidenced by the decreases in blood triglyceride, apolipoprotein B-48, and apolipoprotein B-100 levels following a mixed meal [43,44]. Clinical evidence indicates that the ability of DPP4 inhibitors to ameliorate plasma dyslipidemia is likely to contribute to the cardiovascular benefits [3]. We therefore speculate that DPP4 may participate in the development and progression of CAD partly through its impact on lipid metabolism.

### Study limitations

Study limitations should be considered. First, the small numbers of participants limited the power to prove differences and relationships. Secondly, although the relationship between plasma DPP4 activities and atherosclerotic plaque stenosis and plaque length analyzed by QCA in all CAD patients was significant, the molecular examinations combined with the intravascular ultrasound combined and optimal coherence tomography was not designed to study causality in patients., Third, blood DPP4 activity is not coronary-specific. The widespread expression of DPP4 in blood vessels, myeloid stem cells/progenitor cells, and myocardium has been reported [3]. It is very difficult to separate DPP4 activities from different tissues (myocardium, bone, fat, etc.) and arteries (the cerebral artery, peripheral artery, carotid artery, etc.). Fourth, over 50% of the nonCAD subjects with hypertension or diabetes were not suitable to include in the healthy control group. Fifth, CAD patients with several cardiac diseases as mentioned in the Methods section were excluded. It is unclear how their inclusion or exclusion would influence the present results. In addition, the indexes of actual and prior metabolic controls in our study are lacking. Here, we have also no data showing difference in waist girth or in waist-to-hip ration between the different our study populations. Further studies will be required to investigate these issues.

Indeed, DPP4 activity is increased in proinflammatory states including obesity, diabetes mellitus, and atherosclerosis [18,19]. Accumulating evidence from genetic and pharmacological interventions targeted toward DPP4 proteolytic function in animal models of disease seems to indicate that salutary effects including in heart failure and in totality seem to suggest the possibility of beneficial cardiovascular effects in patients. However, recent large scar randomized clinical trials focused on cardiovascular events in high-risk patients with type 2 DM on best drug therapy seem to show no significant differences compared with placebo with regards to the composite end point of stroke, MI, and cardiovascular death during a long-term follow-up period [45,46]. Experimental evidence indicates that the noncatalytic function of DPP4 (including its roles as a binding to several extracellular matrix proteins including adenosine deaminase and co-stimulatory molecule) in humans can serves to larger role for DPP4 outside of its catalytic function [3]. Future basic and clinical studies will be required to target toward the relative contribution of the non-enzymatic versus enzymatic molecular function in metabolic and inflammatory cardiovascular diseases and address the heart failure safety signals and demonstrate a beneficial effect of this class in cardiovascular complications associated with diabetes.

In conclusion, the results of the present study demonstrated that increased blood DPP4 levels are positively and independently associated with CAD, even without DM. Although a large-scale longitudinal clinical study is needed, our findings suggest that DPP4 exhibits a therapeutic target for atherosclerosis-based cardiovascular disease and that the monitoring of blood DPP4 protein and activities would be useful for the assessment of the risk of the cardiovascular disease.
